# The impact of the timing of mNGS-guided antibiotic adjustment on clinical outcomes in ICU patients with severe community-acquired pneumonia: a retrospective study

**DOI:** 10.1186/s12941-026-00848-5

**Published:** 2026-01-18

**Authors:** Yong Sun, Kai Guo, Jing Tang, Junjie Zhao, Xiaojing Zhang, Youqin Yan, Lingmin Yuan, Yi Zhang, Canhu Qiu, Jian Luo, Juan Chen, Honglong Fang

**Affiliations:** 1 Zhejiang Chinese Medical University, Hangzhou, 310053 Zhejiang China; 2 Emergency and Critical Care Center, Department of Emergency Medicine, Zhejiang Provincial People’s Hospital (Affiliated People’s Hospital, Hangzhou Medical College), Hangzhou, 310014 Zhejiang China; 3 WillingMed Technology Beijing Co., Ltd, Beijing, 101103 China; 4 Department of Critical Care Medicine, People’s Hospital of Changshan County, Quzhou, 324200 Zhejiang China; 5 Department of Critical Care Medicine, Longyou County People’s Hospital, Quzhou, 324499 Zhejiang China; 6 Department of Critical Care Medicine, QuZhou KeCheng People’s Hospital, Quzhou, 324000 Zhejiang China; 7 Department of Critical Care Medicine, Jiangshan People’s Hospital, Quzhou, 324199 Zhejiang China; 8 Department of Critical Care Medicine, The Quzhou Affiliated Hospital of Wenzhou Medical University, Quzhou People’s Hospital, Quzhou, 324000 Zhejiang China; 9 Department of Clinical Laboratory, The Quzhou Affiliated Hospital of Wenzhou Medical University, Quzhou People’s Hospital, Quzhou, 324000 Zhejiang China

**Keywords:** Severe community-acquired pneumonia, Antibiotic adjustment timing, Metagenomic next-generation sequencing, Clinical outcomes

## Abstract

**Background:**

Severe community-acquired pneumonia (SCAP) remains a major cause of intensive care unit (ICU) admission and mortality. Prompt pathogen identification and timely administration of appropriate antimicrobial therapy are essential for improving patient outcomes. Although metagenomic next-generation sequencing (mNGS) enables rapid pathogen detection, the prognostic impact of the timing of mNGS-guided antibiotic adjustment remains unclear.

**Methods:**

We conducted a multicenter retrospective study of ICU patients diagnosed with SCAP who underwent both bronchoalveolar lavage fluid (BALF) mNGS and conventional microbiological tests (CMTs). Patients were categorized into early (≤ 72 h) and late (> 72 h) antibiotic adjustment groups based on the interval from ICU admission to the time of antibiotic adjustment guided by mNGS results. Subgroup analyses were performed according to immune status.

**Results:**

In our study, mNGS significantly outperformed conventional microbiological tests (CMTs) in pathogen detection (92.70% vs. 57.18%, *P* < 0.001), with a particularly higher yield for mixed infections (51.63% vs. 19.14%, *P* < 0.001). Early mNGS-guided antibiotic adjustment was associated with a significantly reduced 28-day mortality compared to late adjustment (41.98% vs. 53.76%, *P* = 0.037). Furthermore, multivariate logistic regression analysis confirmed early adjustment as an independent protective factor for 28-day mortality (adjusted OR = 0.44, 95% CI: 0.23–0.83, *P* = 0.011). In the immunocompromised subgroup, early mNGS-guided adjustment was associated with significantly lower 28-day mortality than late adjustment (39.29% vs. 60.00%, *P* = 0.029), with a significant interaction observed between timing and immune status (*P* = 0.042).

**Conclusion:**

Early mNGS-guided antibiotic adjustment is associated with improved survival among ICU patients with SCAP. This benefit is more pronounced in immunocompromised patients, underscoring the importance of early mNGS application to guide antimicrobial decision-making in this vulnerable population.

## Introduction

Severe community-acquired pneumonia (SCAP) is a major cause of intensive care unit (ICU) admissions, characterized by rapid clinical deterioration and 28-day mortality rates of 30–50% [1, 2]. Timely identification of pathogens and initiation of appropriate antimicrobial therapy are critical to improving patient outcomes [3]. Traditionally, clinicians initiate empirical antibiotic therapy and subsequently adjust treatment based on the results of conventional microbiological tests (CMTs). These tests include microbial cultures, polymerase chain reaction (PCR), and serological assays for pathogen-specific antibodies (such as Mycoplasma pneumoniae, Chlamydia pneumoniae, and Legionella pneumophila), as well as fungal antigen or antibody detection (such as Galactomannan and (1,3)-β-D-glucan tests). However, CMTs exhibit limited sensitivity (50–60%) and often entail delays in providing clinically actionable results, thereby postponing the initiation of targeted antimicrobial therapy [4]. These delays often hinder timely treatment, especially for atypical or fastidious pathogens [5]. This issue is especially critical in immunocompromised patients, who are particularly susceptible to atypical and polymicrobial infections [6, 7].

Metagenomic next-generation sequencing (mNGS) has recently emerged as a promising tool for the etiological diagnosis of infectious diseases [8]. Unlike CMTs, which have limited sensitivity and often require days to yield results, mNGS provides rapid, high-throughput, and unbiased detection of a wide range of pathogens such as bacteria, viruses, fungi, and parasites in a single assay, with results typically available within 24–48 h [9]. This rapid turnaround time is critical in the ICU, where SCAP patients often experience rapid clinical deterioration and high mortality rates. Multiple studies have demonstrated that mNGS achieves a higher diagnostic yield in pneumonia cases compared to conventional testing and is particularly effective in detecting pathogens missed by standard methods, especially in cases involving mixed infections, atypical organisms, or immunocompromised hosts [10–12]. Despite its diagnostic advantages, the clinical impact of the timing of mNGS-guided antibiotic adjustment remains insufficiently explored, particularly in high-risk populations such as immunocompromised patients.

Building on our previous multicenter study [6, 13], which demonstrated that BALF mNGS significantly improved pathogen detection and guided more effective antimicrobial therapy in immunocompromised ICU patients with SCAP, this retrospective study aimed to further investigate the association between the timing of mNGS-guided antibiotic adjustment and clinical outcomes. Specifically, we assessed whether early adjustment (≤ 72 h) reduces 28-day mortality and the duration of mechanical ventilation, with a particular focus on immunocompromised patients.

## Method

### Patients

Between May 2021 and May 2025, a total of 472 ICU patients with SCAP were retrospectively reviewed from five hospitals in China, including People’s Hospital of Quzhou, People’s Hospital of Quzhou Kecheng, People’s Hospital of Changshan, People’s Hospital of Jiangshan, and People’s Hospital of Longyou. While mNGS is frequently employed for complex cases such as SCAP in our ICU, it remains outside routine clinical practice due to its high cost. In this study, mNGS testing was performed when considered clinically indicated by ICU physicians, with all samples collected exclusively from bronchoalveolar lavage fluid (BALF).

SCAP was defined according to the Infectious Diseases Society of America/American Thoracic Society (IDSA/ATS) guidelines [14], requiring either one major criterion: (1) tracheal intubation with mechanical ventilation; (2) septic shock requiring vasopressors after adequate fluid resuscitation or at least three minor criteria: (1) respiratory rate (RR) ≥ 30 breaths/min; (2) oxygenation index ≤ 250 mmHg (1 mmHg = 0.133 kPa); (3) multilobar infiltrates; (4) altered mental status (e.g., confusion or disorientation); (5) blood urea nitrogen (BUN) ≥ 7.14 mmol/L; (6) systolic blood pressure (SBP) < 90 mmHg requiring fluid resuscitation.

The inclusion criteria were: (1) meeting the diagnostic criteria for SCAP; (2) age ≥ 18 years; (3) underwent both CMTs and mNGS testing simultaneously; and (4) received empirical antimicrobial therapy after ICU admission [15].

The exclusion criteria were: (1) incomplete clinical data; and (2) ICU stay < 72 h.

Patients were categorized into early (≤ 72 h) and late (> 72 h) antibiotic adjustment groups based on the interval from ICU admission to the time of antibiotic adjustment guided by mNGS results. The 72-hour threshold was selected in accordance with clinical practice guidelines and previous studies, which identify the 48–72-hour window as a critical period for reassessing empirical antibiotic regimens and implementing targeted therapy, particularly in critically ill or immunocompromised patients [14, 16, 17]. The no-adjustment group included patients whose empirical antibiotic regimens remained unchanged despite receipt of the mNGS report. This lack of adjustment could be attributed to two different scenarios: (1) Appropriate no-adjustment, in which the empirical regimen adequately covered the pathogens identified by mNGS and no modification was necessary; (2) Inappropriate no-adjustment, in which mNGS either failed to detect a clinically relevant pathogen or identified a pathogen not covered by the empirical therapy, yet no modification was made.

To further evaluate the impact of mNGS-guided antibiotic adjustment timing on clinical outcomes in SCAP patients with different immune statuses, patients were stratified into two subgroups: immunocompromised and immunocompetent. Immunocompromised status was defined according to previously published criteria [18, 19] as the presence of at least one of the following risk factors:

1. Hematologic malignancy;
2. History of hematopoietic stem cell or solid organ transplantation;
3. Recent neutropenia or receipt of cytotoxic chemotherapy within the past 3 months;
4. Prolonged corticosteroid therapy (equivalent to ≥ 0.3 mg/kg/day of prednisone for ≥ 3 weeks);
5. Use of disease-modifying antirheumatic drugs, biological immunomodulators, or other immunosuppressive agents.

### Samples collection and conventional Microbiological tests

After ruling out contraindications, bronchoscopy was performed by trained physicians under sterile conditions to collect BALF samples, which were used for both mNGS and conventional microbiological tests (CMTs). Simultaneously, blood, sputum, and other relevant specimens were collected for CMTs. CMTs included BALF culture, sputum culture, blood culture, quantitative polymerase chain reaction (qPCR), T-cell spot test (T-SPOT), GeneXpert, (1,3)-β-D-glucan test (G test), and Galactomannan test (GM test). qPCR was primarily used for viral detection and identification of specific pathogens. T-SPOT and GeneXpert were used to detect *Mycobacterium tuberculosis*. The G test, GM test, and qPCR were used to detect or differentiate invasive fungal infections.

### mNGS procedure

DNA/RNA extraction: BALF samples were centrifuged at 12,075 × g at 4 °C for 5 min. For all included patients, both DNA and RNA sequencing were routinely performed as part of the mNGS workflow. For each sample, 500 µL of supernatant was used to extract genomic DNA using the PathoXtract^®^ WYXM03202S Universal Pathogen Enrichment Extraction Kit (WillingMed, Beijing, China), and RNA was extracted using the PathoXtract^®^ Virus DNA/RNA Isolation Kit (WYXM03009S, WillingMed Corp, Beijing, China), following the manufacturer’s instructions.

Library construction and sequencing: For DNA-only pathogen detection, the Illumina^®^ DNA Prep (M) Tagmentation Kit (20018705; Illumina, San Diego, USA) was used for library preparation. For co-detection of DNA and RNA pathogens, RNA was reverse transcribed into complementary DNA (cDNA) using the SuperScript^®^ Double-Stranded cDNA Synthesis Kit (11917020, Invitrogen), then mixed with the DNA for library construction. Library quality was assessed using a Qubit fluorescence quantification system (Thermo Fisher) and an Agilent 2100 Bioanalyzer (Agilent Technologies). Sequencing was performed on a NextSeq™ 550Dx sequencer (Illumina, San Diego, USA), with each sample generating at least 20 million reads.

Sequencing data were automatically processed to generate a detection report. FASTQ-formatted sequencing data were processed using Trimmomatic v0.40 [20] to remove low-quality reads, contaminants, high-copy repeats, and short-read sequences. High-quality reads were then aligned to the human reference genome GRCh37 (hg19) using Bowtie2 v2.4.3 [21] to eliminate host-derived sequences. The remaining non-human reads were aligned to a comprehensive reference database containing over 24,000 pathogens using Kraken2 v2.1.0 [22] for pathogen identification. Microbial genome databases were downloaded from GenBank (http://ftp.ncbi.nlm.nih.gov/genomes/genbank/).

### Concordance evaluation

The consistency of the results between mNGS and CMTs for clinical diagnosis was comprehensively assessed by two experienced clinicians with over 10 years of ICU experience, leading to a final diagnosis based on the comprehensive results. The diagnosis took into account the patients’ clinical characteristics, results of CMTs, results of mNGS, laboratory data, and other relevant factors. Additionally, chest computed tomography (CT) and other imaging modalities are also employed to assist in the overall diagnostic process. To highlight the differences between mNGS and CMTs in detecting pathogenic microorganisms, we defined the following criteria and used a composite pie chart to visually represent the results:

1. Complete match: The pathogenic microorganisms identified by mNGS and CMTs were in complete consistent.
2. Partial match: The pathogenic microorganisms identified by mNGS and CMTs were partially consistent, the mNGS and/or CMTs identified additional pathogenic microorganisms.
3. Mismatch: The pathogenic microorganisms identified by mNGS and CMTs were completely inconsistent.

### Criteria for a positive mNGS results

The criteria for a clinically positive mNGS result in this study were as follows: for viruses, reads per ten million (RPTM) ≥ 3; for bacteria and fungi, RPTM ≥ 10. A threshold of ≥ 1 read was applied for specific pathogens such as nontuberculous *mycobacteria*, *Legionella*, and similar organisms [23–25].

### Evaluation of clinical impact of mNGS results

In this study, the clinical impact of mNGS results on antibiotic decision-making was independently evaluated by two senior ICU physicians, each with over 10 years of clinical experience. Discrepancies between evaluators were resolved through discussion and consensus.

The therapeutic consequences of mNGS were categorized into five types based on changes in antibiotic management.

1. Appropriate targeted therapy: The empirical regimen did not cover the pathogen(s) identified by mNGS. Treatment was modified specifically to target the newly identified organisms, such as by adding or switching to antibiotics, antifungals, antivirals, or antiparasitic agents based on mNGS findings.
2. Antibiotic escalation: The empirical regimen already covered the pathogen(s) identified by mNGS, but the antimicrobial spectrum was broadened—such as by adding new agents or switching to broad-spectrum antibiotics—due to clinical concerns, including worsening symptoms, suspected resistance, or mixed infections, rather than direct mismatch between therapy and detected pathogens.
3. Antibiotic de-escalation: The regimen was simplified based on mNGS results, including dose reduction, discontinuation of unnecessary agents, or substitution of broad-spectrum antibiotics with narrower-spectrum alternatives.
4. No change: The empirical regimen sufficiently covered the pathogens reported by mNGS; therefore, no modification was made.
5. Misleading adjustment: mNGS results either failed to detect the true pathogen(s) or misidentified irrelevant organisms, resulting in inappropriate escalation, overtreatment, or delayed effective therapy.

### Statistical analysis

Statistical analyses were performed using SPSS v29.0 and R v4.5.0, while OriginPro 2025 were used exclusively for data visualization and figure preparation. The Kolmogorov–Smirnov test was used to assess the distribution normality of continuous variables. Variables with normal distribution were expressed as mean ± standard deviation and compared using Student’s t-test; non-normally distributed variables were presented as median (interquartile range, IQR) and analyzed using the Mann–Whitney U test. Categorical variables were presented as n (%) and compared using the Chi-square test or Fisher’s exact test. Kaplan–Meier survival curves and log-rank tests were used to assess survival differences. Multivariate logistic regression was used to identify independent predictors of 28-day mortality, with adjustment for age, white blood cell, C-reactive protein, procalcitonin, platelets, hemoglobin, creatinine, D-dimer, lactate, APACHE II score, and immune status. An interaction term (early adjustment × immunocompromised status) was included in the model to evaluate effect modification. A two-tailed *P*-value < 0.05 was considered statistically significant.

**Fig. 1:**
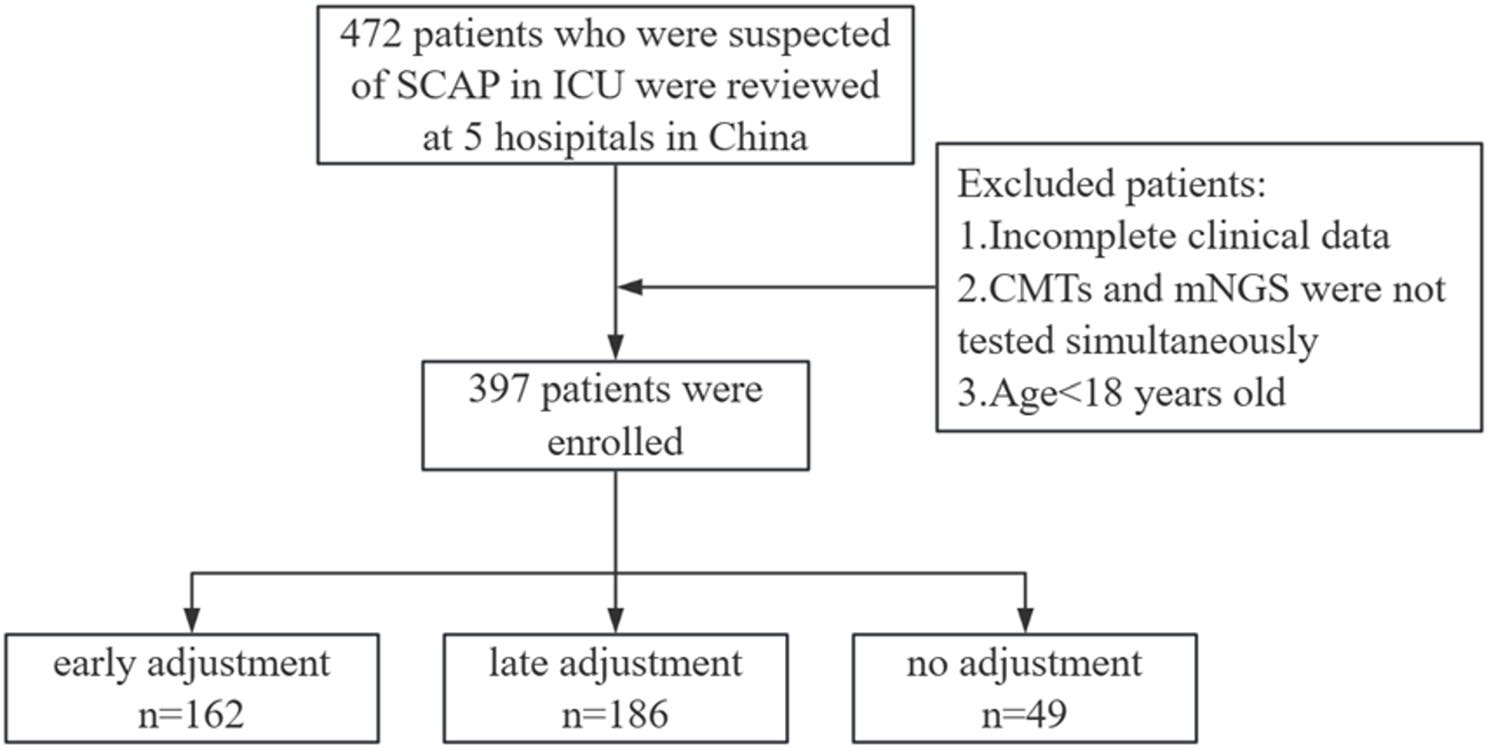
Study flowchart of patient inclusion and group classification

## Results

### Clinical characteristics of patients

A total of 472 ICU patients with suspected SCAP were initially screened across five hospitals in China between May 2021 and May 2025. After screening, 397 patients were included in this study (Fig. 1). Based on the timing of mNGS-guided antibiotic adjustment, patients were categorized into an early adjustment group (≤ 72 h, *n* = 162), a late adjustment group (> 72 h, *n* = 186), and a no adjustment group (*n* = 49). Baseline clinical characteristics of the early and late adjustment groups are shown in Table 1.

Compared with the late adjustment group, patients in the early adjustment group showed no significant differences in age, sex distribution, or the proportion of immunocompromised individuals (all *P* > 0.05). The prevalence of common comorbidities, including hypertension, diabetes, cardiovascular disease, cerebrovascular disease, and chronic respiratory disease, was also comparable between the two groups (all *P* > 0.05). In laboratory tests, the early adjustment group had higher white blood cell counts (8.70 vs. 7.20 × 10⁹/L, *P* = 0.017), C-reactive protein (CRP) levels (62.60 vs. 47.54 mg/L, *P* = 0.022), and D-dimer levels (4.66 vs. 2.36 mg/L, *P* = 0.004), but lower platelet counts (119.80 vs. 168.40 × 10⁹/L, *P* = 0.001), hemoglobin levels (104 vs. 115 g/L, *P* = 0.003), and serum creatinine (62.35 vs. 72.80 µmol/L, *P* = 0.003). Other laboratory parameters, including procalcitonin, neutrophil count, total bilirubin, and lactate levels, did not show significant differences between the two groups (all *P* > 0.05). Regarding disease severity, the early adjustment group had higher APACHE II scores (20 vs. 17, *P* = 0.031), while SOFA scores remained similar (12 vs. 12, *P* = 0.627). The proportion of patients requiring mechanical ventilation was also similar between groups (97.53% vs. 95.16%, *P* = 0.245), although the duration of mechanical ventilation was significantly shorter in the early adjustment group (7 [3–12] vs. 10 [4–17] days; *P* = 0.028). Notably, 28-day mortality was significantly lower in the early adjustment group than in the late adjustment group (41.98% vs. 53.76%, *P* = 0.037). In parallel, The median laboratory turnaround time from mNGS sample collection to result reporting was 24 h (IQR, 18–36) in the early adjustment group and 26 h (IQR, 19–38) in the late adjustment group, with no statistically significant difference between groups (*P* = 0.382).

**Table 1:** Baseline clinical characteristics of patients grouped by the timing of mNGS-guided antibiotic adjustment

	Early adjustment group (*n* = 162)	Late adjustment group(*n* = 186)	*P*-value
Age, years, median (IQR)	69 (58–79)	67(61–74)	0.147
Sex, n (%)			
Male	95(58.64%)	113(60.75%)	0.689
Female	67(41.36%)	73(39.25%)	
Immunocompromised, n (%)	56(34.73%)	65(35.11%)	0.941
Underlying diseases, n (%)			
Hypertension	45 (27.78%)	55 (29.57%)	0.713
Diabetes	24 (14.81%)	26 (13.98%)	0.824
Cardiovascular disease	11 (6.79%)	12 (6.45%)	0.899
Cerebrovascular disease	10 (6.17%)	8 (4.30%)	0.432
Respiratory disease	38 (23.46%)	41 (22.04%)	0.754
Clinical laboratory tests, median (IQR)			
WBC, ×10^9^/L	8.70(5.10–11.90)	7.20 (4.90–9.90)	0.017
PCT, ng/ml	2.09(0.02–12.87)	1.47(0.02–10.53)	0.069
CRP, mg/L	62.60(9.32–152.0)	47.54(7.47-118.38)	0.022
Platelets, ×10^9^/L	119.80(55.70-177.50)	168.40(108.50-237.50)	0.001
Hemoglobin, g/L	104(95.00-116.00)	115.00(97.25–127.00)	0.003
Neutrophil count, ×10^9^/L	5.46(3.06–9.53)	5.64(3.39–7.86)	0.683
Bilirubin, µmol/L	11.94 (7.47–16.53)	11.18 (6.30–15.60)	0.264
Creatinine, µmol/L	62.35(47.13–77.48)	72.80(52.67–88.17)	0.003
D-dimer, mg/L	4.66 (0.50–7.89)	2.36 (0.00–5.84)	0.004
Lactic acid, mmol/L	1.81 (1.42–2.31)	1.99 (1.14–2.61)	0.537
Disease severity			
APACHE-Ⅱ, median (IQR)	20(12–26)	17(9–23)	0.024
SOFA, median (IQR)	12(8–15)	12(6–14)	0.627
ICU treatment			
MV n (%)	158(97.53%)	177(95.16%)	0.245
Length of MV days, median (IQR)	7(3–12)	10(4–17)	0.028
28-day mortality n (%)	68(41.98%)	100(53.76%)	0.037
Time from mNGS sampling to result reporting, h, median (IQR)	24 (18–36)	26 (19–38)	0.382

*WBC* white blood cell count,* PCT* procalcitonin,* CRP* C-reactive protein,* APACHE II* Acute Physiology and Chronic Health Evaluation II,* MV* mechanical ventilation,* IQR* interquartile range,* ICU* intensive care unit

### Evaluation of the concordance of the mNGS and CMTs results

**Fig. 2:**
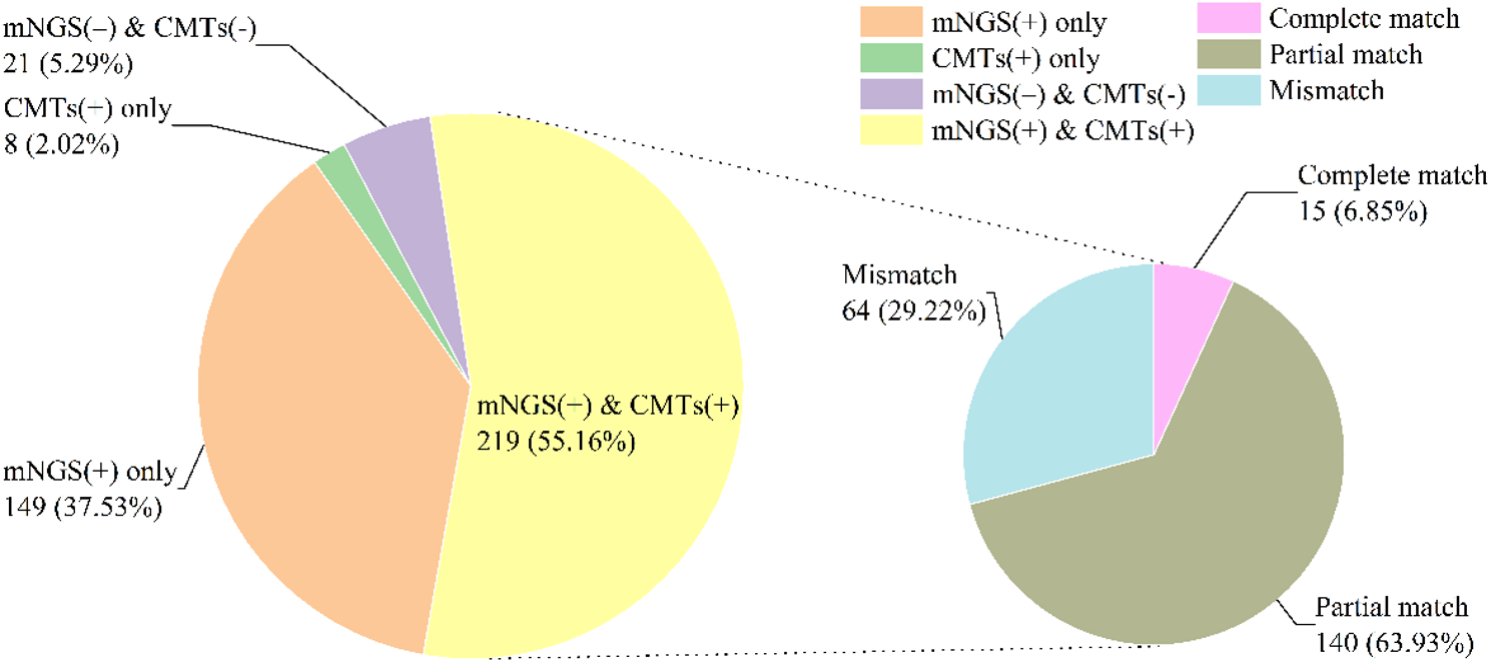
Concordance of microorganism detection between mNGS and CMTs

Among all patients, Dual positivity, defined as positive results from both mNGS and CMTs, was observed in 219 patients (55.16%), while dual negativity was identified in 21 patients (5.29%). mNGS-only positivity was present in 149 cases (37.53%), whereas CMT-only positivity was detected in 8 cases (2.02%). Among the 219 patients with dual positivity, 15 cases (6.85%) showed complete match between mNGS and CMTs, 140 cases (63.93%) showed partial match, and 64 cases (29.22%) showed mismatch in detected microorganisms (Fig. 2).

### Pathogen detection performance of mNGS vs. CMTs

**Fig. 3:**
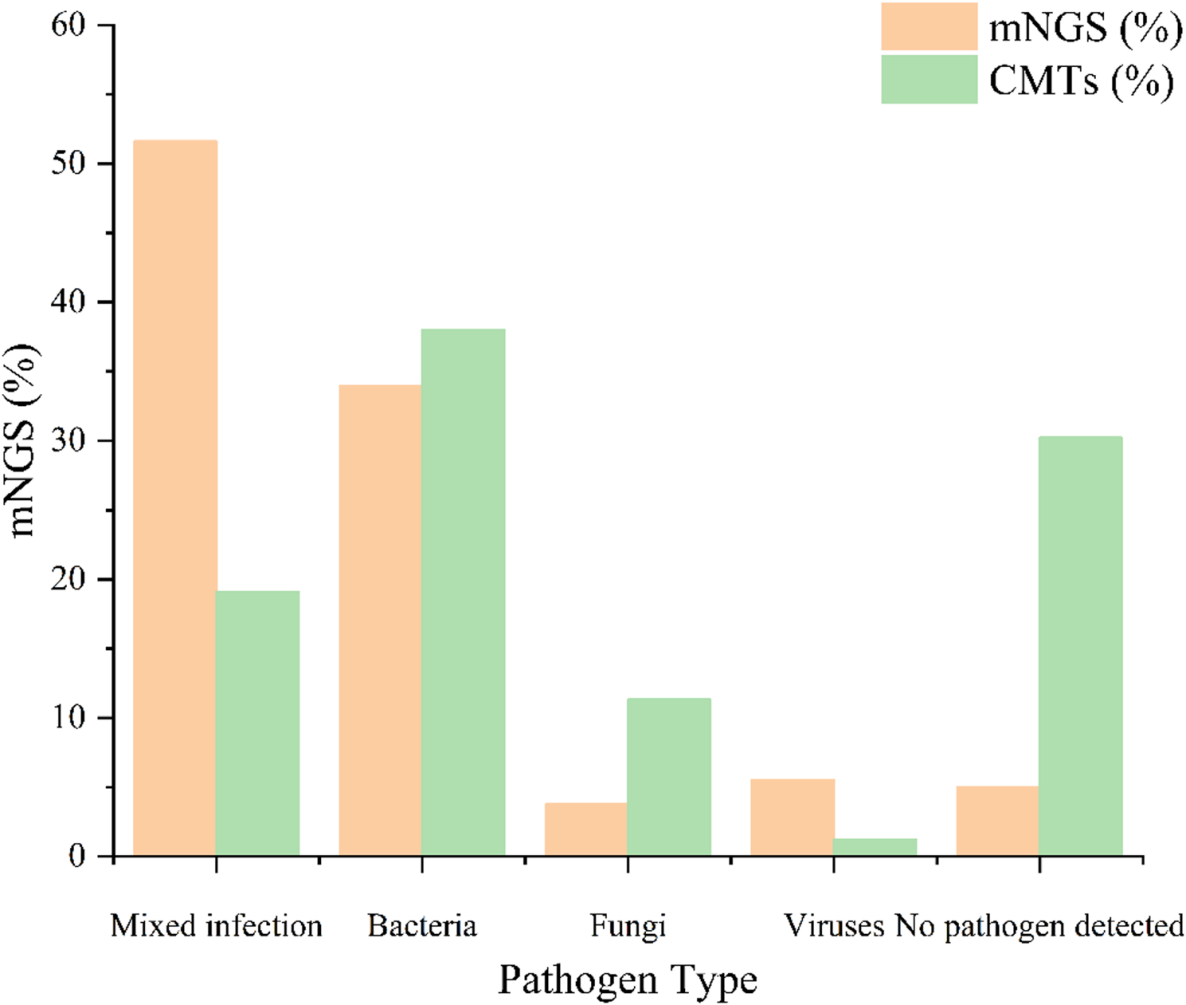
Comparison of infection types detected by mNGS and conventional microbiological tests

mNGS demonstrated a significantly higher pathogen detection rate compared to conventional microbiological tests (CMTs) (92.70% vs. 57.18%, *P* < 0.001). It also identified mixed infections in more than half of the patients, substantially outperforming CMTs (51.63% vs. 19.14%, *P* < 0.001) (Fig. 3).

**Fig. 4:**
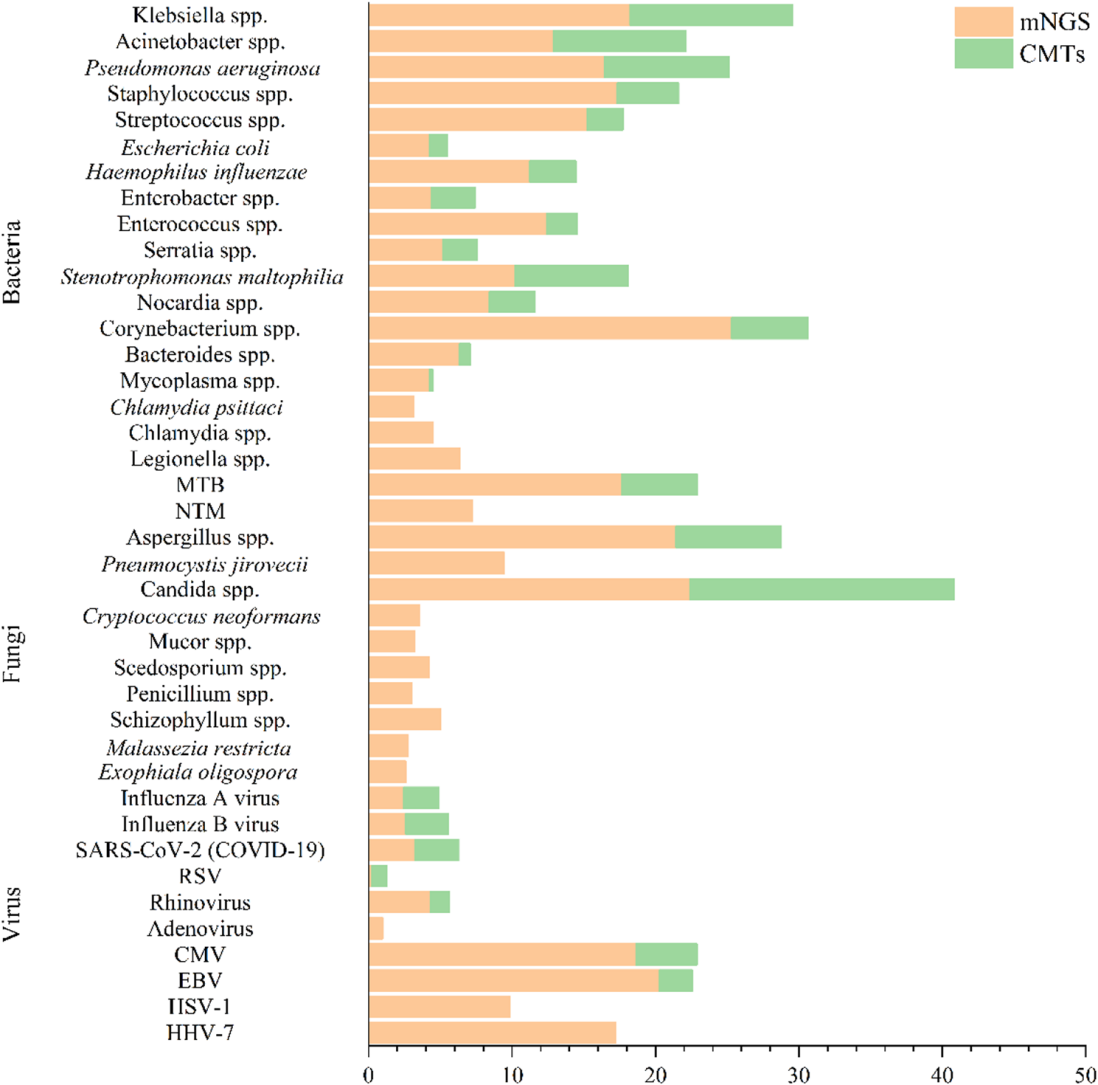
Distribution of microorganism detection rates by mNGS and conventional methods

Species-level analysis further highlighted the broader detection capability of mNGS (Fig. 4). Among bacterial pathogens, Acinetobacter species, Klebsiella species, and *Pseudomonas aeruginosa* were more frequently detected by mNGS. For fungal pathogens, the mNGS group exhibited higher detection rates of Candida species, Aspergillus species, Penicillium species and *Pneumocystis jirovecii*. Moreover, mNGS identified a wider spectrum of respiratory and latent viruses, including Epstein–Barr virus (EBV), cytomegalovirus (CMV), human herpesvirus 7 (HHV-7), and adenovirus, which were rarely detected by CMTs. In addition, atypical or fastidious organisms, such as Mycoplasma species, *Chlamydia psittaci*, nontuberculous mycobacteria (NTM), and Legionella species, were almost exclusively identified through mNGS.

### Antibiotic adjustment strategies by timing

**Fig. 5:**
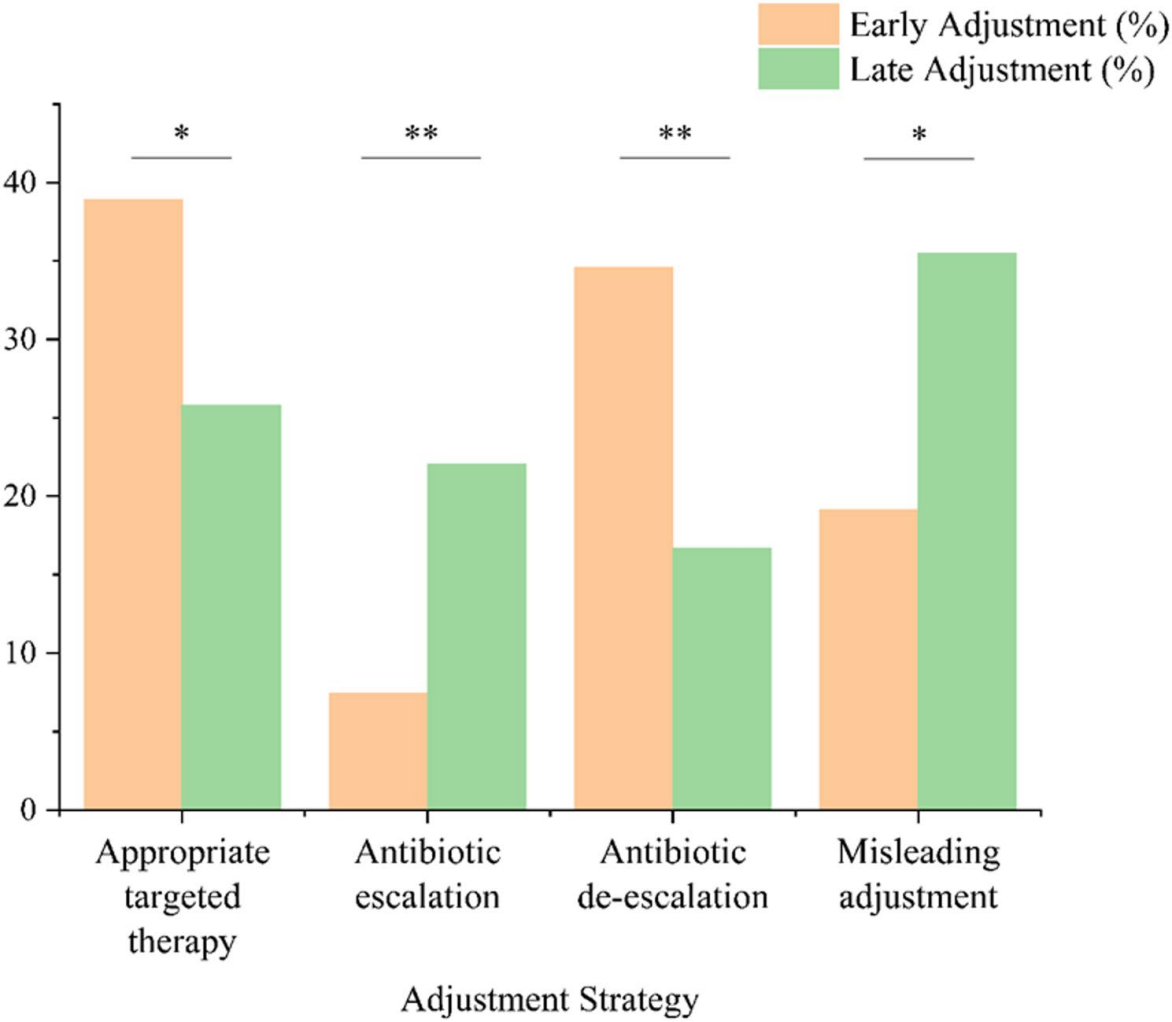
Distribution of antibiotic adjustment strategies between early and late mngs-guided adjustment groups *P < 0.05 **P < 0.001

Antibiotic adjustment strategies were compared between early and late adjustment groups to assess differences in treatment patterns (Fig. 5). De-escalation (34.57% [56/162] vs. 16.67% [31/186], *P* < 0.001) and appropriate targeted therapy (38.89% [63/162] vs. 25.81% [48/186], *P* = 0.013) were significantly more frequent in the early adjustment group. In contrast, empirical escalation (22.04% [41/186] vs. 7.41% [12/162], *P* < 0.001) and misleading adjustment (35.48% [66/186] vs. 19.14% [31/162], *P* = 0.001) were more commonly observed in the late adjustment group.

### Early antibiotic adjustment reduced patients 28-day mortality

**Fig. 6:**
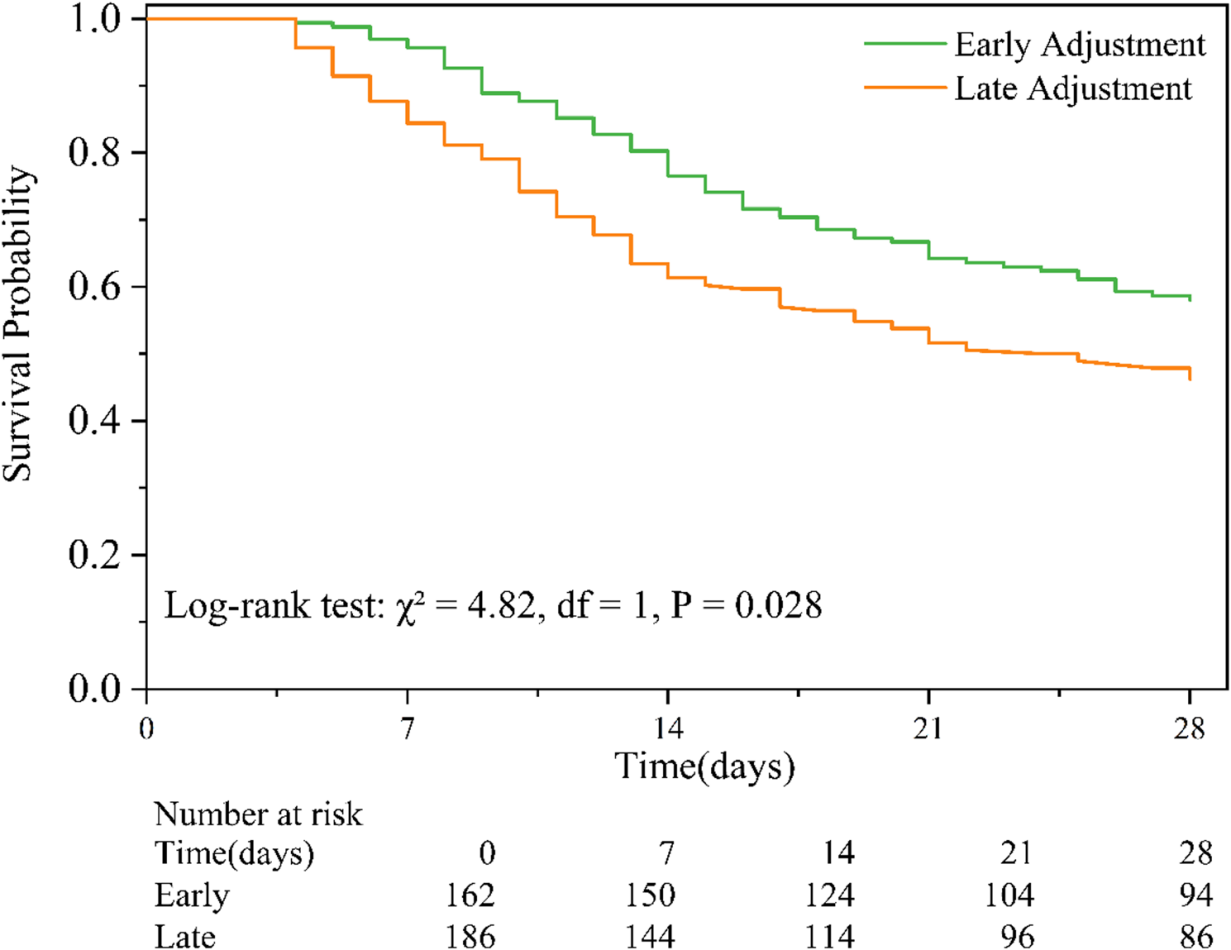
Kaplan-Meier survival curves comparing early and late mNGS-guided antibiotic adjustment

Among the 348 patients who underwent mNGS-guided antibiotic adjustment, early adjustment was associated with a significantly lower 28-day mortality compared to late adjustment (41.98% [68/162] vs. 53.76% [100/186], *P* = 0.028). Kaplan–Meier survival analysis confirmed this benefit, showing significantly improved survival rate in the early adjustment group (log-rank test: χ² = 4.82, df = 1, *P* = 0.028; Fig. 6).

**Fig. 7:**
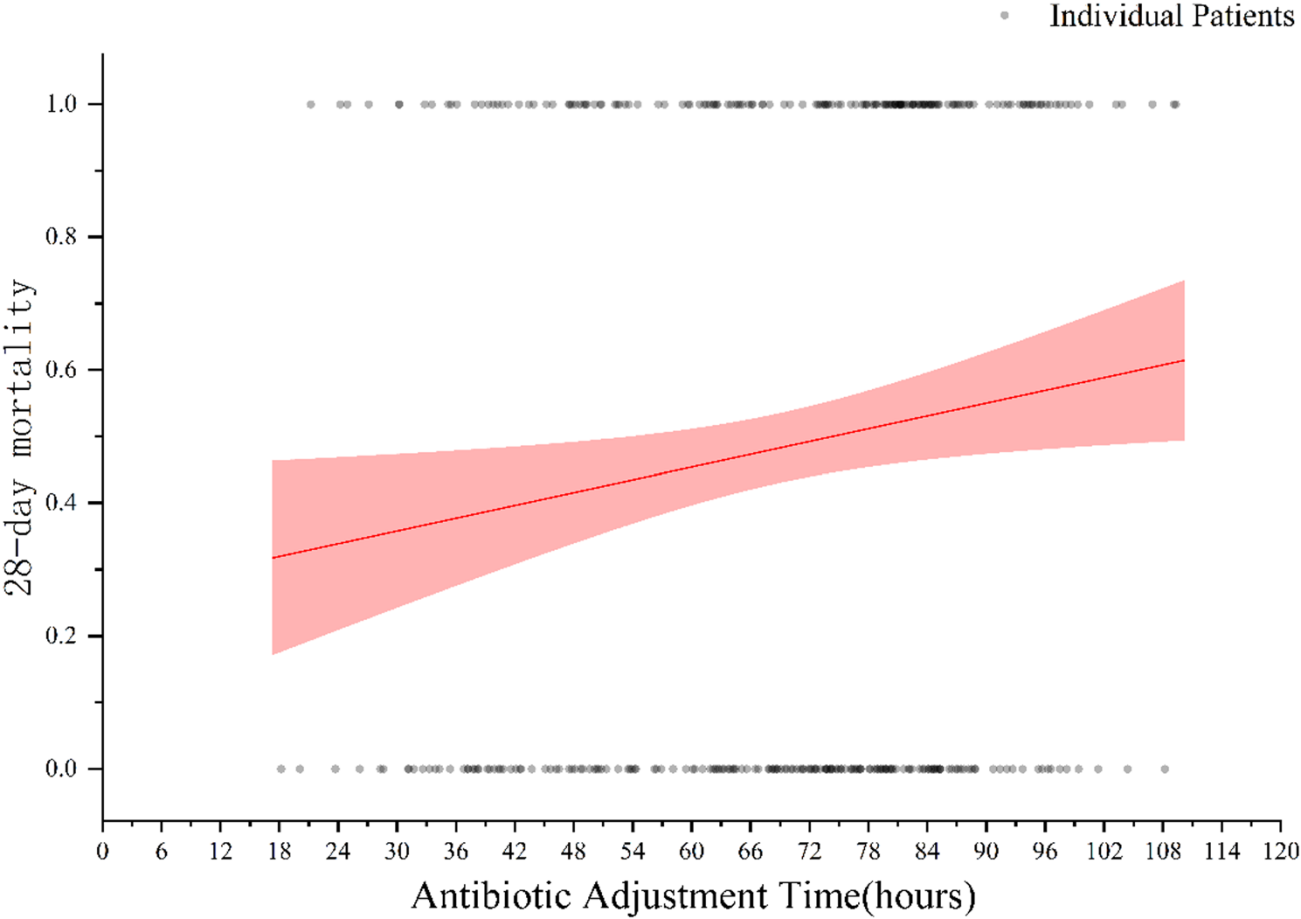
Association between adjustment timing and predicted 28-day mortality

To evaluate the impact of adjustment timing on mortality risk, we performed a logistic regression analysis. Logistic regression analysis demonstrated a positive association between antibiotic adjustment time and 28-day mortality (Fig. 7). In the overall cohort, each one-hour delay in mNGS-guided antibiotic adjustment was associated with a 1.3% increase in the risk of 28-day mortality (OR = 1.013, 95% CI: 1.002–1.024). Among patients whose adjustment occurred beyond 72 h, each additional hour of delay was associated with a 4.0% increase in mortality risk (OR = 1.040, 95% CI: 1.003–1.078).

To identify factors associated with patient prognosis, we compared baseline clinical characteristics between survivors (*n* = 180) and non-survivors (*n* = 168) (Table 2). There were no significant differences in age, sex, or the proportion of immunocompromised individuals, nor in the prevalence of common comorbidities such as hypertension, diabetes, cardiovascular disease, cerebrovascular disease, and respiratory disease (all *P* > 0.05). However, non-survivors exhibited significantly elevated levels of inflammatory and organ dysfunction markers, including white blood cell count (9.30 vs. 7.40 × 10⁹/L, *P* = 0.042), procalcitonin (2.60 vs. 0.75 ng/mL, *P* = 0.009), C-reactive protein (72.01 vs. 44.21 mg/L, *P* = 0.031), D-dimer (5.36 vs. 2.10 mg/L, *P* = 0.004), serum creatinine (75.80 vs. 64.35 µmol/L, *P* = 0.048), and lactate (2.25 vs. 1.65 mmol/L, *P* = 0.008), while hemoglobin levels were significantly lower in non-survivors (99 vs. 116 g/L, *P* = 0.023). Regarding disease severity, non-survivors had higher APACHE II scores (18 vs. 16, *P* = 0.031), although SOFA scores were similar between the two groups (13 vs. 12, *P* = 0.517). The need for mechanical ventilation was high in both groups but was more frequent in non-survivors (98.81% vs. 93.89%, *P* = 0.033), who also had a significantly longer duration of mechanical ventilation (11 [5–16] vs. 7 [4–13] days, *P* = 0.026).

**Fig. 8:**
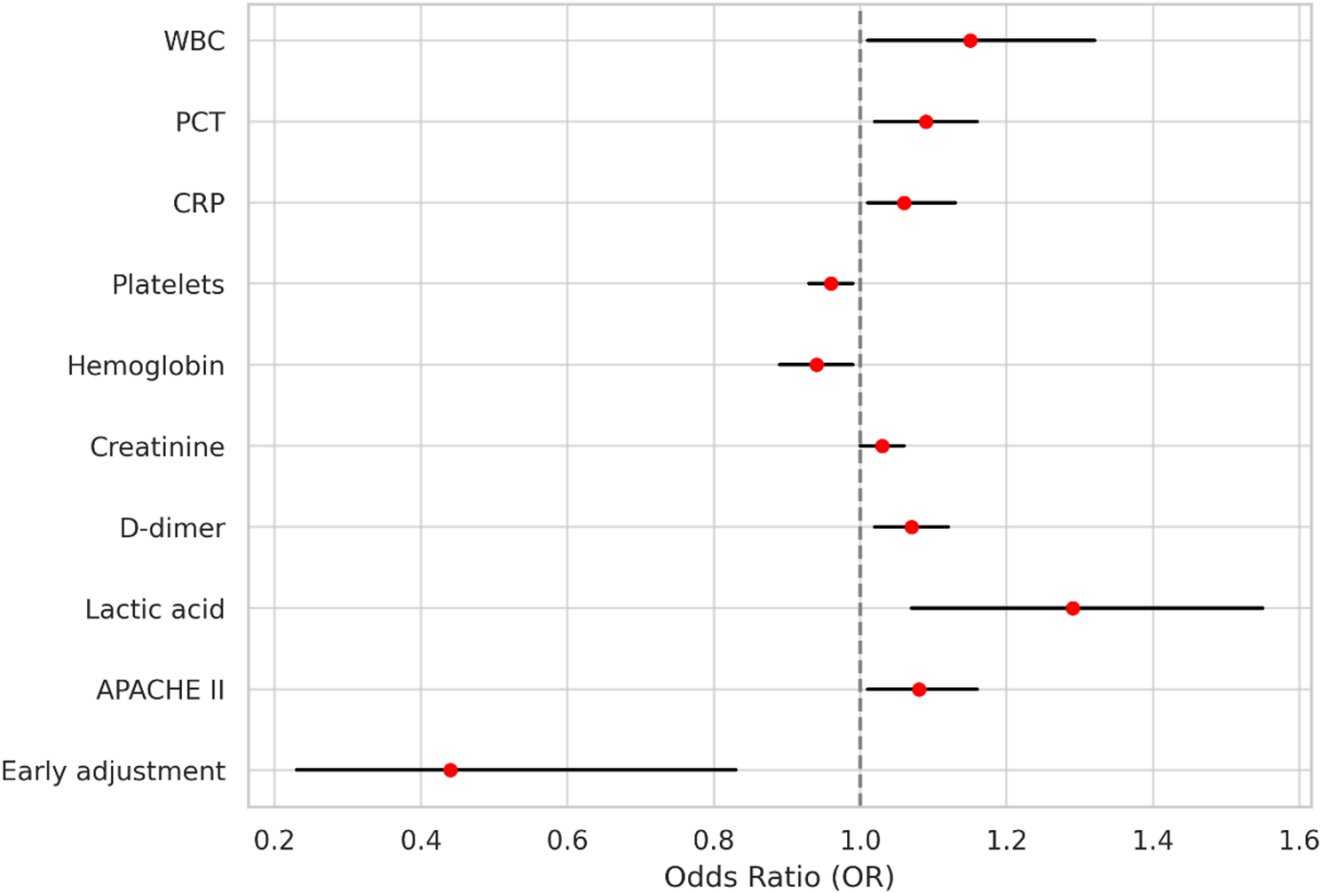
Forest plot of multivariate logistic regression identifying predictors of 28-day mortality

Variables with a P-value < 0.1 in univariate analysis were included in the multivariate logistic regression model. Multivariate logistic regression analysis further confirmed that early adjustment was independently associated with reduced 28-day mortality (adjusted OR = 0.44, 95% CI: 0.23–0.83, *P* = 0.011; Fig. 8).

**Table 2:** Comparison of baseline clinical characteristics between survivors and non-survivors

	Survivors (*n* = 180)	Non-survivors (*n* = 168)	*P*-value
Age, years, median (IQR)	69 (58–79)	71 (61–81)	0.147
Sex, n (%)			0.266
Male	102 (56.67%)	106 (63.10%)	
Female	78 (43.33%)	62 (36.90%)	
Immunocompromised, n (%)	60 (33.33%)	61 (36.31%)	0.638
Underlying diseases, n (%)			
Hypertension	48 (26.67%)	52 (30.95%)	0.445
Diabetes	22 (12.22%)	28 (16.67%)	0.304
Cardiovascular disease	10 (5.56%)	13 (7.74%)	0.547
Cerebrovascular disease	7 (3.89%)	11 (6.55%)	0.381
Respiratory disease	34 (18.89%)	45 (26.79%)	0.103
Clinical laboratory tests, median (IQR)			
WBC, ×10^9^/L	7.40(4.70–9.80)	9.30 (5.60–12.50)	0.042
PCT, ng/ml	0.75(0.01–6.51)	2.60(0.60–8.53)	0.009
CRP, mg/L	44.21(6.32-105.11)	72.01(16.51-162.38)	0.031
Platelets, ×10^9^/L	170.10(105.00-177.50)	108.40(48.50-160.50)	0.065
Hemoglobin, g/L	116(99.00-127.00)	99(91.25–110.00)	0.023
Neutrophil count, ×10^9^/L	5.46(3.06–9.53)	5.64(3.39–7.86)	0.683
Bilirubin, µmol/L	10.91 (6.17–15.53)	12.60 (8.30–17.60)	0.098
Creatinine, µmol/L	64.35(47.13–77.48)	75.80(52.67–92.17)	0.048
D-dimer, mg/L	2.10 (0.00–5.89)	5.36 (0.00–5.84)	0.004
Lactic acid, mmol/L	1.65 (1.02–2.31)	2.25 (1.14–2.90)	0.008
Disease severity			
APACHE-Ⅱ, median (IQR)	16 (8–21)	18 (9–23)	0.031
SOFA, median (IQR)	12 (8–15)	13 (6–19)	0.517
ICU treatment			
MV n (%)	169 (93.89%)	166 (98.81%)	0.033
Length of MV days, median (IQR)	7 (4–13)	11 (5–16)	0.026
Early adjustment n (%)	94 (52.22%)	68 (40.48%)	0.037

### Impact of immune status on mNGS-Guided antibiotic adjustment and outcomes

**Fig. 9:**
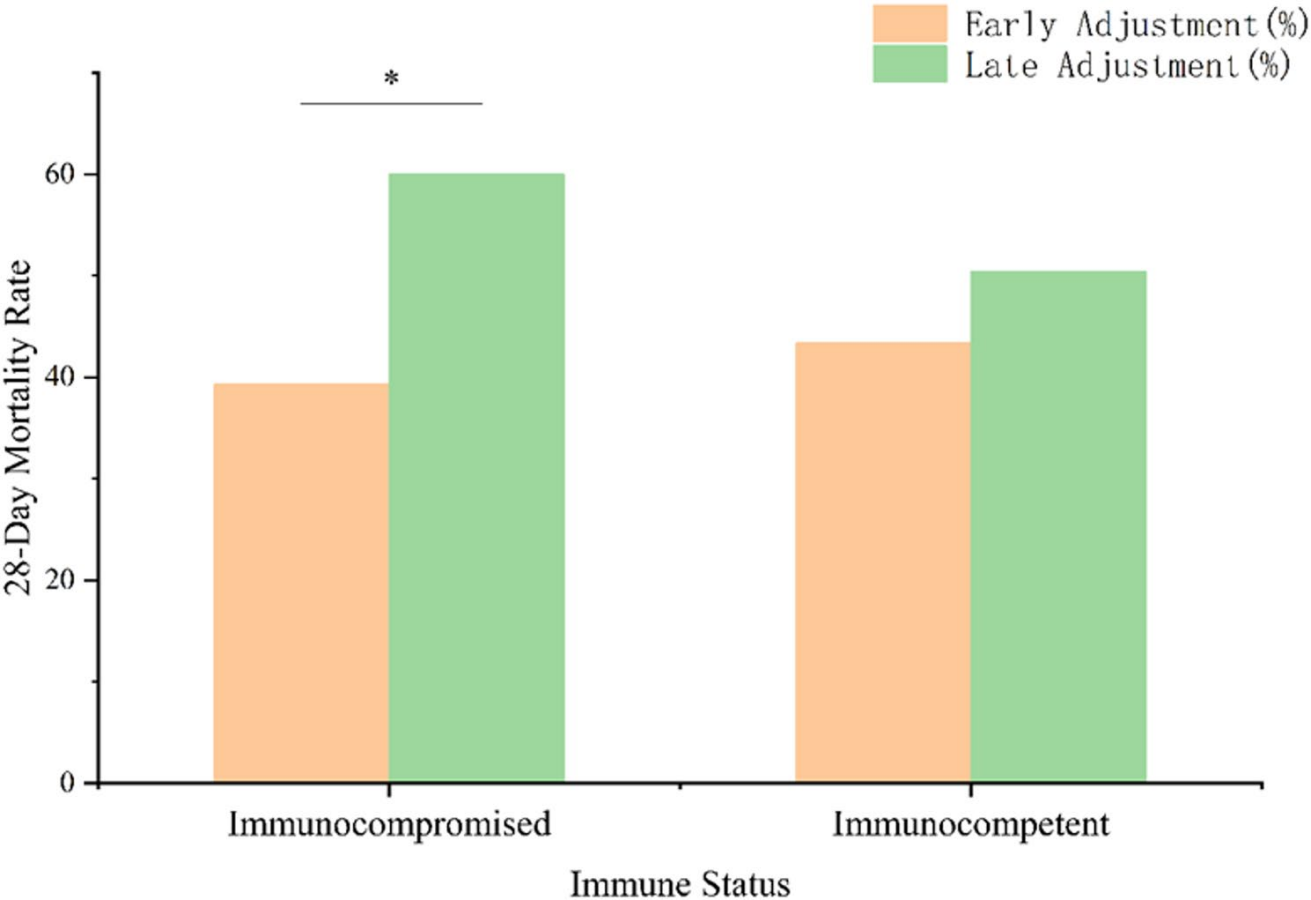
Comparison of 28-day mortality by antibiotic adjustment timing and immune status. *P < 0.05

As shown in Fig. 9, we further investigated whether the effect of mNGS-guided antibiotic adjustment timing varied according to immune status. Among immunocompromised patients (*n* = 142), early adjustment was associated with a significantly lower 28-day mortality compared to late adjustment (39.29% [22/56] vs. 60.00% [39/65], *P* = 0.029). A similar trend was observed among immunocompetent patients (*n* = 255), although the difference did not reach statistical significance (43.40% [46/106] vs. 50.41% [61/121], *P* = 0.351).

A statistically significant interaction was observed between adjustment timing and immune status (P for interaction = 0.042), suggesting that the clinical effect of early antibiotic adjustment differed according to patients’ immune function.

### Clinical outcomes in the no adjustment group

Among patients who did not receive mNGS-guided antibiotic adjustment (*n* = 49), the overall 28-day mortality rate was 51.02% (25/49), which was comparable to that of the late adjustment group. When stratified, patients in the Appropriate No Adjustment subgroup (*n* = 25), whose empirical antibiotic regimens adequately covered the pathogens identified by mNGS, had a lower 28-day mortality rate of 32.00% (8/25). In contrast, patients in the Inappropriate No Adjustment subgroup (*n* = 24), in whom mNGS either failed to detect clinically relevant pathogens or identified organisms not covered by the empirical therapy, had a substantially higher mortality rate of 70.83% (17/24).

## Discussion

This multicenter retrospective study demonstrates that early mNGS-guided antibiotic adjustment is associated with improved survival in ICU patients with SCAP. Patients who received antibiotic adjustment within 72 h of ICU admission had significantly lower 28-day mortality. Subgroup analysis further revealed that immunocompromised patients derived greater benefit from early adjustment, and immune status significantly modified the relationship between adjustment timing and outcomes.

mNGS has emerged as a powerful diagnostic tool in infectious disease management due to its ability to detect a comprehensive range of pathogens, including bacteria, viruses, fungi, and parasites, without requiring prior assumptions about the causative agent [26]. A growing body of evidence has demonstrated that mNGS outperforms CMTs in terms of sensitivity, pathogen breadth, and turnaround time [27], this is consistent with our findings, with median mNGS turnaround times of 26 h in the early adjustment group and 28 h in the late adjustment group. In our study, mNGS showed a significantly higher diagnostic yield compared to CMTs in ICU patients with severe community-acquired pneumonia (SCAP) (92.71% vs. 57.18%), particularly for mixed infections (51.64% vs. 19.14%). Moreover, mNGS uniquely identified atypical or fastidious pathogens such as Mycoplasma spp., *Chlamydia psittaci*, nontuberculous mycobacteria, and Legionella spp., which are frequently missed by traditional methods. Species-level analysis further reinforced the broader detection capability of mNGS. Among bacterial pathogens, *Acinetobacter baumannii*, Klebsiella species, and *Pseudomonas aeruginosa* were more frequently detected by mNGS. For fungal infections, mNGS demonstrated higher detection rates of Candida, Aspergillus, Penicillium species and *Pneumocystis jirovecii*. Additionally, mNGS identified a wider range of respiratory and latent viruses, including Epstein–Barr virus (EBV), cytomegalovirus (CMV), human herpesvirus 7 (HHV-7), and adenovirus, which were rarely captured by conventional methods (Fig. 4). These results are consistent with findings reported in previous studies [28, 29]. Beyond its diagnostic superiority, the timeliness of mNGS results is crucial in optimizing antimicrobial therapy. Prompt identification of causative pathogens within the first 48–72 h of ICU admission provide a critical window for intervention [30, 31]. Our findings show patients in the early adjustment group were more likely to receive targeted therapy (38.89% vs. 25.81%, *P* = 0.013) and de-escalation (34.57% vs. 16.67%, *P* < 0.001), while those in the late adjustment group were more often subjected to empirical escalation and inappropriate treatment strategies (Fig. 5). These findings suggest that early mNGS-guided adjustment not only improves the accuracy of pathogen-directed therapy but also promotes antimicrobial stewardship by reducing unnecessary broad-spectrum antibiotic use. This may help mitigate antibiotic-related adverse effects and resistance, ultimately contributing to better clinical outcomes and improving patient prognosis.

SCAP remains one of the most serious infectious diseases in ICU, with reported mortality rates exceeding 30–50% despite timely empirical treatment [2]. The early phase of SCAP is frequently characterized by rapid clinical deterioration and a narrow therapeutic window, highlighting the importance of timely pathogen identification and appropriate antibiotic administration [1, 32]. Early initiation of targeted antimicrobial therapy is essential to effective SCAP management, as delays in aligning treatment with the causative pathogen can result in prolonged inflammation, higher rates of organ dysfunction, and increased mortality. Our findings indicate that early mNGS-guided antibiotic adjustment was significantly associated with lower 28-day mortality compared to late adjustment (41.98% vs. 53.76%, *P* = 0.028). This survival benefit remained robust after adjusting for potential confounders in multivariable logistic regression analysis, with early adjustment confirmed as an independent protective factor (adjusted OR = 0.44, 95% CI: 0.23–0.83, *P* = 0.011). Other protective variables included higher platelet counts and hemoglobin levels, consistent with previous studies [33, 34]. However, among these, the timing of antibiotic adjustment demonstrated the strongest association with reduced mortality. To further elucidate the time-dependent effect of adjustment, we analyzed the relationship between timing and mortality risk. The results showed that each one-hour delay in adjustment was associated with a 1.3% increase in the risk of death (OR = 1.013, 95% CI: 1.002–1.024). Among patients whose adjustment occurred beyond 72 h, each additional hour of delay was associated with a 4.0% increase in mortality risk (OR = 1.040, 95% CI: 1.003–1.078). This time–risk relationship mirrors the findings from prior sepsis research. For example, Im Y et al. reported that in patients with septic shock, administration of antibiotics within 1 h of sepsis recognition significantly reduced hospital mortality, with a 35% increase in mortality risk for every hour of delay [35]. Importantly, prior studies have shown that mNGS typically provides clinically actionable results within 24–48 h [36], and the laboratory turnaround time observed in our cohort was consistent with this range. This finding suggests that the observed differences in antimicrobial adjustment timing were unlikely to be driven primarily by laboratory-related delays. Instead, mNGS may facilitate timely pathogen identification within the first 48–72 h after ICU admission, enabling clinicians to intervene within a limited therapeutic window through earlier targeted therapy, ultimately improving survival outcomes and potentially shortening ICU stay in patients with SCAP. This concept is further supported by previous sepsis studies demonstrating that each hour of delay in initiating effective antimicrobial therapy is associated with a significant increase in mortality [37, 38].

In addition to adjustment timing, our analysis revealed that the specific nature of antibiotic adjustment strategies also significantly impacted clinical outcomes. As shown in Fig. 5, early mNGS-guided antibiotic adjustment was more often associated with targeted therapy and antibiotic de-escalation, while late adjustment was more commonly associated with empirical escalation or inappropriate therapy. The observed differences in antibiotic adjustment strategies may reflect the timeliness of microbiological data. When mNGS results are available early, they align more closely with the evolving clinical picture, allowing clinicians to make pathogen-directed decisions such as de-escalation or targeted therapy with greater confidence. In contrast, late adjustments may occur after clinical deterioration, prompting empirical escalation due to clinical urgency rather than microbiological evidence. Delayed adjustment in this setting may therefore partly reflect delayed clinical decision-making rather than laboratory-related factors. These findings are closely aligned with the principles of antimicrobial stewardship (AMS) [39], which advocate for timely and individualized adjustments based on pathogen-directed data [40, 41]. Notably, the present study was designed to evaluate mNGS-guided diagnostic and decision-making strategies rather than the clinical efficacy of specific antimicrobial agents. Although mNGS frequently identified pathogens such as cytomegalovirus, Epstein–Barr virus, and Aspergillus species, we did not perform a drug-level outcome analysis to assess the impact of individual antiviral or antifungal therapies. Therefore, the observed survival benefit should be interpreted as reflecting the overall effect of timely, pathogen-informed antimicrobial adjustment rather than the effect of any specific agent.

Immunocompromised patients are particularly vulnerable to atypical, viral, or polymicrobial infections and often present diagnostic and therapeutic challenges due to blunted inflammatory responses and low pathogen yields from conventional testing [42]. In our study, subgroup analysis revealed that this population derived significantly greater benefit from early mNGS-guided antibiotic adjustment, with a markedly lower 28-day mortality compared to late adjustment (39.29% [22/56] vs. 60.00% [39/65], *P* = 0.029). In contrast, among immunocompetent patients, although early adjustment was associated with a lower mortality rate (43.40% [46/106] vs. 50.41% [61/121]), the difference did not reach statistical significance (*P* = 0.351). This discrepancy may reflect differences in pathogen profiles, disease severity, or other clinical factors. For instance, immunocompetent patients may have a higher prevalence of typical bacterial pathogens, which are more likely to be adequately covered by empirical therapy, thus reducing the incremental benefit of early mNGS-guided adjustments. Additionally, immunocompetent patients in our cohort had slightly lower APACHE II scores (median 16 vs. 18, *P* = 0.031), suggesting potentially less severe disease, which could diminish the impact of early intervention. Other factors, such as variations in host immune response or the presence of fewer polymicrobial infections in immunocompetent patients, may also contribute to the attenuated effect. Further analysis of pathogen types and clinical characteristics in this subgroup is needed to clarify these differences. A significant interaction term (P for interaction = 0.042) suggests that immune status modifies the effect of antibiotic adjustment timing on clinical outcomes, underscoring the importance of immune function as a key determinant of who benefits most from early mNGS-guided therapy. Nevertheless, given the limited sample size within individual immunocompromised subgroups, our findings should be interpreted at the population level rather than attributed to any single immunocompromised phenotype. Further studies are required to better define the immunocompromised subgroups that benefit most from early mNGS-guided therapy.

In addition, we evaluated patients who received no adjustment despite the availability of mNGS results. Notably, patients in the inappropriate no-adjustment subgroup had the highest observed mortality (70.83%), significantly exceeding that of the early adjustment group and even the late adjustment group. This finding highlights the potential harm of underutilizing actionable diagnostic data and emphasizes that the benefits of mNGS rely not only on timely testing but also on prompt and appropriate clinical application. The consistent mortality gradient observed across the early, late, and no-adjustment groups strengthens the conclusion that the combination of mNGS with timely decision-making is essential for maximizing its clinical utility.

## Limitation

Despite its diagnostic advantages, mNGS also has some limitations. Its high sensitivity may lead to the detection of non-pathogenic colonizers or contaminants, particularly in specimens like BALF. This necessitates expert interpretation within the clinical context to avoid misleading therapy, as observed in a subset of our patients. Furthermore, challenges remain regarding standardization of sequencing platforms, reporting criteria, and cost-effectiveness in real-world settings. While our findings support the adoption of early mNGS-guided strategies, they stem from a retrospective design that may be subject to residual confounding and selection bias. In addition, we did not evaluate the effect of individual antimicrobial agents, and drug-specific outcome analyses were beyond the scope of this study. Although the immunocompromised subgroup was clearly defined, it encompassed diverse etiologies of immune suppression, which may have influenced treatment responses and outcomes. Prospective randomized trials are needed to validate these results, assess cost-benefit ratios, and explore the integration of mNGS with host-response biomarkers and decision-support algorithms to enhance personalized care in the ICU.

## Conclusions

Early antibiotic adjustment guided by mNGS was associated with significantly improved survival in ICU patients with severe community-acquired pneumonia, with the benefit being especially pronounced in immunocompromised individuals. These findings underscore the importance of timely pathogen identification and support the early integration of mNGS into the diagnostic and therapeutic workflow for SCAP—particularly in patients with impaired immune function. Further prospective studies are warranted to confirm these results and to explore the broader clinical implications of mNGS-guided therapy.

## Data Availability

The datasets used and/or analyzed during the current study are available from the corresponding author on reasonable request.

## References

[1] Niederman MS, Torres A. Severe community-acquired pneumonia. Eur Respir Rev. 2022;31:220123. 10.1183/16000617.0123-2022. 36517046 10.1183/16000617.0123-2022 PMC9879347

[2] Nair GB, Niederman MS. Updates on community acquired pneumonia management in the ICU. Pharmacol Ther. 2021;217:107663. 10.1016/j.pharmthera.2020.107663. 32805298 10.1016/j.pharmthera.2020.107663 PMC7428725

[3] Messacar K, Parker SK, Todd JK, et al. Implementation of rapid molecular infectious disease diagnostics: the role of diagnostic and antimicrobial stewardship. J Clin Microbiol. 2017;55:715–23. 10.1128/JCM.02264-16. 28031432 10.1128/JCM.02264-16 PMC5328439

[4] Torres A, Niederman MS, Chastre J, et al. International ERS/ESICM/ESCMID/ALAT guidelines for the management of hospital-acquired pneumonia and ventilator-associated pneumonia: guidelines for the management of hospital-acquired pneumonia (HAP)/ventilator-associated pneumonia (VAP) of the European respiratory society (ERS), European society of intensive care medicine (ESICM), European society of clinical microbiology and infectious diseases (ESCMID) and Asociación Latinoamericana Del Tórax (ALAT). Eur Respir J. 2017;50:1700582. 10.1183/13993003.00582-2017. 28890434 10.1183/13993003.00582-2017

[5] Wu X, Sun T, He H et al. Effect of metagenomic Next-Generation sequencing on clinical outcomes of patients with severe Community-Acquired pneumonia in ICU: A Multicenter Randomized Controlled Trial. 10.1016/j.chest.2024.07.144 39067508

[6] Zhao J, Sun Y, Tang J, et al. The clinical application of metagenomic next-generation sequencing in immunocompromised patients with severe respiratory infections in the ICU. Respir Res. 2024;25:360. 10.1186/s12931-024-02991-z. 39369191 10.1186/s12931-024-02991-z PMC11453054

[7] Azoulay E, Métais M, Lemiale V, et al. Outcomes in immunocompromised patients with acute hypoxemic respiratory failure treated by high-flow nasal oxygen. Intensive Care Med. 2025;51:731–41. 10.1007/s00134-025-07890-5. 40261380 10.1007/s00134-025-07890-5

[8] Gu W, Miller S, Chiu CY. Clinical metagenomic Next-Generation sequencing for pathogen detection. Annu Rev Pathol Mech Dis. 2019;14:319–38. 10.1146/annurev-pathmechdis-012418-012751. 10.1146/annurev-pathmechdis-012418-012751 PMC6345613 30355154

[9] Lin T, Tu X, Zhao J, et al. Microbiological diagnostic performance of metagenomic next-generation sequencing compared with conventional culture for patients with community-acquired pneumonia. Front Cell Infect Microbiol. 2023;13:1136588. 10.3389/fcimb.2023.1136588. 37009509 10.3389/fcimb.2023.1136588 PMC10061305

[10] Parize P, Muth E, Richaud C, et al. Untargeted next-generation sequencing-based first-line diagnosis of infection in immunocompromised adults: a multicentre, blinded, prospective study. Clin Microbiol Infect. 2017;23:574. .e1-574.e6. 10.1016/j.cmi.2017.02.006 28192237

[11] Sun T, Wu X, Cai Y, et al. Metagenomic Next-Generation sequencing for pathogenic diagnosis and antibiotic management of severe Community-Acquired pneumonia in immunocompromised adults. Front Cell Infect Microbiol. 2021;11:661589. 10.3389/fcimb.2021.661589. 34141628 10.3389/fcimb.2021.661589 PMC8204719

[12] Qu J, Zhang J, Chen Y, et al. Aetiology of severe community acquired pneumonia in adults identified by combined detection methods: a multi-centre prospective study in China. Emerg Microbes Infections. 2022;11:556–66. 10.1080/22221751.2022.2035194. 10.1080/22221751.2022.2035194 PMC8843176 35081880

[13] Zhao J, Zhuge R, Hu B, et al. Clinical impact of Bronchoalveolar lavage fluid metagenomic next-generation sequencing in immunocompromised patients with severe community-acquired pneumonia in ICU: a multicenter retrospective study. Infection Published Online First: 23 April. 2025. 10.1007/s15010-025-02520-0. 10.1007/s15010-025-02520-0 PMC12460554 40268850

[14] Olson G, Davis AM. Diagnosis and treatment of adults with Community-Acquired pneumonia. JAMA. 2020;323:885. 10.1001/jama.2019.21118. 32027358 10.1001/jama.2019.21118

[15] Martin-Loeches I, Reyes LF, Rodriguez A. Severe community-acquired pneumonia (sCAP): advances in management and future directions. *Thorax*. 2025;thorax-2024-222296. 10.1136/thorax-2024-222296 10.1136/thorax-2024-222296 40360263

[16] Kalil AC, Metersky ML, Klompas M, et al. Management of adults with Hospital-acquired and Ventilator-associated pneumonia: 2016 clinical practice guidelines by the infectious diseases society of America and the American thoracic society. Clin Infect Dis. 2016;63:e61–111. 10.1093/cid/ciw353. 27418577 10.1093/cid/ciw353 PMC4981759

[17] Bassetti M, Giacobbe DR, Magnasco L, et al. Antibiotic strategies for severe Community-Acquired pneumonia. Semin Respir Crit Care Med. 2024;45:187–99. 10.1055/s-0043-1778641. 38301712 10.1055/s-0043-1778641

[18] Kreitmann L, Helms J, Martin-Loeches I, et al. ICU-acquired infections in immunocompromised patients. Intensive Care Med. 2024;50:332–49. 10.1007/s00134-023-07295-2. 38197931 10.1007/s00134-023-07295-2

[19] Ramirez JA, Musher DM, Evans SE, et al. Treatment of Community-Acquired pneumonia in immunocompromised adults. Chest. 2020;158:1896–911. 10.1016/j.chest.2020.05.598. 32561442 10.1016/j.chest.2020.05.598 PMC7297164

[20] Bolger AM, Lohse M, Usadel B. Trimmomatic: a flexible trimmer for illumina sequence data. Bioinformatics. 2014;30:2114–20. 10.1093/bioinformatics/btu170. 24695404 10.1093/bioinformatics/btu170 PMC4103590

[21] Langmead B, Salzberg SL. Fast gapped-read alignment with bowtie 2. Nat Methods. 2012;9:357–9. 10.1038/nmeth.1923. 22388286 10.1038/nmeth.1923 PMC3322381

[22] Wood DE, Lu J, Langmead B. Improved metagenomic analysis with kraken 2. Genome Biol. 2019;20:257. 10.1186/s13059-019-1891-0. 31779668 10.1186/s13059-019-1891-0 PMC6883579

[23] Chen H, Yin Y, Gao H, et al. Clinical utility of In-house metagenomic Next-generation sequencing for the diagnosis of lower respiratory tract infections and analysis of the host immune response. Clin Infect Dis. 2020;71:S416–26. 10.1093/cid/ciaa1516. 33367583 10.1093/cid/ciaa1516

[24] Chen H, Zheng Y, Zhang X, et al. Clinical evaluation of cell-free and cellular metagenomic next-generation sequencing of infected body fluids. J Adv Res. 2024;55:119–29. 10.1016/j.jare.2023.02.018. 36889461 10.1016/j.jare.2023.02.018 PMC10770109

[25] Langelier C, Zinter MS, Kalantar K, et al. Metagenomic sequencing detects respiratory pathogens in hematopoietic cellular transplant patients. Am J Respir Crit Care Med. 2018;197:524–8. 10.1164/rccm.201706-1097LE. 28686513 10.1164/rccm.201706-1097LE PMC5821905

[26] Wilson MR, Sample HA, Zorn KC, et al. Clinical metagenomic sequencing for diagnosis of meningitis and encephalitis. N Engl J Med. 2019;380:2327–40. 10.1056/NEJMoa1803396. 31189036 10.1056/NEJMoa1803396 PMC6764751

[27] Yin Y, Zhu P, Guo Y, et al. Enhancing lower respiratory tract infection diagnosis: implementation and clinical assessment of multiplex PCR-based and hybrid capture-based targeted next-generation sequencing. eBioMedicine. 2024;107:105307. 10.1016/j.ebiom.2024.105307. 39226681 10.1016/j.ebiom.2024.105307 PMC11403251

[28] Di Pasquale MF, Sotgiu G, Gramegna A, et al. Prevalence and etiology of Community-acquired pneumonia in immunocompromised patients. Clin Infect Dis. 2019;68:1482–93. 10.1093/cid/ciy723. 31222287 10.1093/cid/ciy723 PMC6481991

[29] Wu X, Li Y, Zhang M, et al. Etiology of severe Community-Acquired pneumonia in adults based on metagenomic Next-Generation sequencing: A prospective multicenter study. Infect Dis Ther. 2020;9:1003–15. 10.1007/s40121-020-00353-y. 33170499 10.1007/s40121-020-00353-y PMC7652912

[30] Lodise TP, Bonine NG, Ye JM, et al. Development of a bedside tool to predict the probability of drug-resistant pathogens among hospitalized adult patients with gram-negative infections. BMC Infect Dis. 2019;19. 10.1186/s12879-019-4363-y. 10.1186/s12879-019-4363-y PMC6694572 31412809

[31] Umpleby H, Dushianthan A, Catton T et al. Antimicrobial stewardship programmes focused on de-escalation: a narrative review of efficacy and risks.

[32] Chen S, Kang Y, Li D, et al. Diagnostic performance of metagenomic next-generation sequencing for the detection of pathogens in Bronchoalveolar lavage fluid in patients with pulmonary infections: systematic review and meta-analysis. Int J Infect Dis. 2022;122:867–73. 10.1016/j.ijid.2022.07.054. 35907477 10.1016/j.ijid.2022.07.054

[33] Huang S-S, Qiu J-Y, Li S-P, et al. Microbial signatures predictive of short-term prognosis in severe pneumonia. Front Cell Infect Microbiol. 2024;14:1397717. 10.3389/fcimb.2024.1397717. 39157177 10.3389/fcimb.2024.1397717 PMC11327560

[34] Gao Q, Li L, Su T, et al. A single-center, retrospective study of hospitalized patients with lower respiratory tract infections: clinical assessment of metagenomic next-generation sequencing and identification of risk factors in patients. Respir Res. 2024;25:250. 10.1186/s12931-024-02887-y. 38902783 10.1186/s12931-024-02887-y PMC11191188

[35] Im Y, Kang D, Ko R-E, et al. Time-to-antibiotics and clinical outcomes in patients with sepsis and septic shock: a prospective nationwide multicenter cohort study. Crit Care. 2022;26:19. 10.1186/s13054-021-03883-0. 35027073 10.1186/s13054-021-03883-0 PMC8756674

[36] Qian Y-Y, Wang H-Y, Zhou Y, et al. Improving pulmonary infection diagnosis with metagenomic next generation sequencing. Front Cell Infect Microbiol. 2021;10:567615. 10.3389/fcimb.2020.567615. 33585263 10.3389/fcimb.2020.567615 PMC7874146

[37] Sterling SA, Miller WR, Pryor J, et al. The impact of timing of antibiotics on outcomes in severe sepsis and septic shock: A systematic review and Meta-Analysis*. Crit Care Med. 2015;43:1907–15. 10.1097/CCM.0000000000001142. 26121073 10.1097/CCM.0000000000001142 PMC4597314

[38] Sherwin R, Winters ME, Vilke GM, et al. Does early and appropriate antibiotic administration improve mortality in emergency department patients with severe sepsis or septic shock? J Emerg Med. 2017;53:588–95. 10.1016/j.jemermed.2016.12.009. 28916120 10.1016/j.jemermed.2016.12.009

[39] Bodilsen J, D’Alessandris QG, Humphreys H, et al. European society of clinical microbiology and infectious diseases guidelines on diagnosis and treatment of brain abscess in children and adults. Clin Microbiol Infect. 2024;30:66–89. 10.1016/j.cmi.2023.08.016. 37648062 10.1016/j.cmi.2023.08.016

[40] Tamma PD, Miller MA, Cosgrove SE. Rethinking how antibiotics are prescribed: incorporating the 4 moments of antibiotic decision making into clinical practice. JAMA. 2019;321:139. 10.1001/jama.2018.19509. 30589917 10.1001/jama.2018.19509

[41] Pulcini C, Binda F, Lamkang AS, et al. Developing core elements and checklist items for global hospital antimicrobial stewardship programmes: a consensus approach. Clin Microbiol Infect. 2019;25:20–5. 10.1016/j.cmi.2018.03.033. 29625170 10.1016/j.cmi.2018.03.033

[42] Varley CD, Streifel AC, Bair AM, et al. Nontuberculous mycobacterial pulmonary disease in the immunocompromised host. Clin Chest Med. 2023;44:829–38. 10.1016/j.ccm.2023.06.007. 37890919 10.1016/j.ccm.2023.06.007

